# Promoting University Students' Mental Health: A Systematic Literature Review Introducing the 4M-Model of Individual-Level Interventions

**DOI:** 10.3389/fpubh.2021.699030

**Published:** 2021-06-25

**Authors:** Bhavana Nair, Farah Otaki

**Affiliations:** ^1^Guidance & Counseling Office, Student Services & Registration, Mohammed Bin Rashid University of Medicine and Health Sciences (MBRU), Dubai, United Arab Emirates; ^2^Strategy & Institutional Excellence, Mohammed Bin Rashid University of Medicine and Health Sciences (MBRU), Dubai, United Arab Emirates

**Keywords:** mental health, health and well-being, holism, university students, mindfulness, higher education, student support

## Abstract

**Objective:** The purpose of this study is to systematically review recently published individual student-level interventions aimed at alleviating the burden of mental health challenges faced by the students and/ or at equipping them with coping mechanism that will foster their resilience.

**Methods:** This study relied on a systematic literature review. PubMed dataset was used; the search was confined to the following period: July 2016-December 2020.

**Results:** A total of 1,399 records were identified by the electronic search, out of which 40 studies were included in this study. The authors inductively identified four overlapping categories of interventions across all included articles, and coded them as follows: Mindfulness, Movement, Meaning, and Moderator. Accordingly, each study was linked to at least one of four overlapping categories based on the nature of the intervention(s) under investigation, leading to differing assortments of categories.

**Conclusions:** The 4M-Model generated by this study encourages focusing on devising holistic, university-based interventions that embrace the individuality of students to improve their mental health through elements of mindfulness, movement, meaning, and moderator. Through this focused approach, university counselors are enabled to design interventions that address students' physical, psychological, emotional, and social needs.

## Introduction

There has been a positive paradigm shift in the way our world and its citizens are perceiving the concept of mental health. Mental health is a state of well-being that allows individuals to enjoy and maintain relationships as well as handle stress in a healthy manner without compromising on productivity (1).

A large body of literature on tertiary education students highlights the importance of maintaining mental health with evidence relating it to educational attainment and productivity (2), social relationships, engagement on campus, and quality of life (3), and placement performance (4). Poor mental health has also been linked with lower retention within a programme, grade point averages, and graduation rates among university students (5). Counseling, psychoeducation, and mental health services on campuses are no longer deemed as merely supportive but rather an integral component necessary to empower students. These services are integral to help students develop skills such as psychological flexibility (6) which in turn influences mental health (1).

The current generation of university students is vastly different from previous generations, especially in their attitudes and beliefs toward their mental health needs. Well-being is a dynamic concept of interlinked physical, social, and psychological dimensions which is constantly changing depending on intrinsic and extrinsic environments and motivations (7). It is not only the demographics of the current generation of university students that has changed considerably from the past (8), but so have their attitudes and beliefs toward their needs, including mental health (3). This population is considered high risk because most mental health problems are triggered before the age of 24 (9). There is enough evidence to link personal and academic stressors to mental health (10–12). Contemporary tertiary education is striving to attain and maintain cultures of excellence, similar to traditional universities in the past (13). However, there has been a shift to turn modern day campuses into high stakes competitive testing environments with well-intended emphasis on preparing students to become part of the global economy. This change has influenced the context in which modern universities function. There are a set of challenges that contemporary universities face that extend beyond the earlier tertiary educational institutions and there is an assumption that students are coming to college “overwhelmed and more damaged than those of previous years” (14).

Although good citizenship has always been an important foundation of all educational institutions, with the dynamic social landscape that the universities are set within, there seems to be a tendency to lead students to fixate on extrinsic factors such as: results and Grade Point Averages, over intrinsic interest such as innovative learning, and expansion of lateral thinking (13). When the priority is grades, it manifests itself in excessive hours of focused studying, and in negative coping behaviors, such as: inadequate sleep and addictive behaviors, which could potentially affect the well-being of the student. Often, in this pursuit of academic excellence, there is the danger of ignoring the social, emotional, and psychological problems that modern students are now increasingly facing.

There is enough research that indicates that students are experiencing more mental health disorders in contemporary times and are less resilient than students in the past (8), with lower levels of frustration tolerance (15). Anxiety and depression are most prevalent among tertiary students (16). There is a rise in the number of college students with a diagnosable psychological disorder (17) with some students at greater risk than others of experiencing stress and mental health problems (18). There has been also a shift in the severity of the problems by students seeking counseling services over the past decade. It is no longer just presenting challenges of adjustment and individuation (19), or benign hormonal developmental problems associated with the age that prompts students to seek counseling. Students are presenting with severe psychological problems (20) with a sizeable number of them on psychiatric medication to help them function better on campus (15).

A common narrative through an exhaustive body of literature highlights the barriers to seeking help for mental health problems by students on campus due to stigma (21), scepticism about treatment efficacy (22), and a belief that their emotional problems will not be completely understood. This leads to a sense of social isolation as the students restrain from reaching out for help (21). Two contributing factors to inadequate help-seeking are the stigma of having a mental health problem and the personal characteristics of the individual student (20). A fear of negative consequences on academic records (23) is another common barrier among university students. Interestingly, students resist seeking help because they do not perceive their condition to require intervention or do not perceive it as a priority among their other commitments. They also have the tendency to normalize stress as part of university life, expecting it “will go away with time,” and prefer to handle their problems on their own (24).

More recent research indicates that students also rely on informal sources of help-seeking from non-professionals, particularly peer groups (25). Students report having no inhibitions about having open discussions about their mental health problems via social-networking websites (26). This resonates with the network episode model of help-seeking that emphasizes the social network as an integral, contemporary support in enhancing knowledge and attitudes toward seeking help (27). However, there is also a significant increase in the number of students with major psychological problems seeking counseling services on campus (3) challenging the stigma connected with help-seeking. The newer generation's familiarity with psychosocial support services and openness toward seeking them are putting mental health at the core of self-care, much like diet and exercise (26).

Along with rapid social changes and expectations, the dilution of traditional family anchors (that is the changes to family systems which include busy yet isolated lifestyles, social media pressures, a living free from parental influence which is very common to this age group, and forced separation from families in the pursuit of dream destinations for education) all compounding to the considerable degree of stress that students report upon (18). Considering all these transitions, focusing on the support that is available to young people on campus is increasingly becoming a necessity. This is not only a personal benefit for students but a national and international investment that could also result in considerable economic benefit (28) as these students stand to become contributors to the global economy.

A wealth of research exists which highlights the effectiveness of changing organizational factors that influence mental health (29, 30). However, there is limited research on person-centric mental health strategies used in university settings (31). A Systematic Literature Review that was conducted by Fernandez et al. focused on evaluating the effect of setting-based interventions that stimulated and improved the mental health and well-being of university students and employees (32). That review constitutes an asset for universities seeking to adopt setting-based strategies that were proven efficacious. Yet, given the highspeed in which the higher education ecosystem has been evolving, there is an evident need for a more up-to-date review. Also, despite the importance of modifying the environment for it to become more nurturing for university students' mental health, this needs to be in conjunction with embracing the individuality of each student. Accordingly, the purpose of this study is to bridge this gap through providing a review of the literature on recently published individual student-level interventions that aim to alleviate the burden of mental health challenges faced by the students and/or help them with coping mechanisms that will foster their resilience.

## Methods

We conducted a systematic review following the Preferred Reporting Items for Systematic Reviews and Meta-Analyses (PRISMA) guidelines (33). The protocol of the systematic review was published in PROSPERO, a database of prospectively registered systematic reviews in health and social care (CRD42021227862).

### Search Strategy

To complement the work of Fernandez et al., focusing on the recent literature, the search period was confined to July 2016 through December 2020 (32). PubMed database was used. The search strategy used, with its key words and Boolean logic, is available as an online resource. It was structured as follows:

- *Subjects:* student *or* resident.
- *Location:* higher education, university, college, *or* tertiary education.
- *State-of-being*: mental health.
- *Challenges faced by subjects*: psychosocial, anxiety, depression, burnout, stress, peer-pressure, social media pressure, bullying, eating disorder, perfectionism, *or* learning difficulties.
- *Intervention to address the challenges*: psychotherapy, mindfulness, Counseling, support group, yoga, breathing, art therapy, awareness, resilience, gratitude, affirmations, *or* peer-Counseling.

Pure qualitative studies were excluded. We included all quantitative studies, so long as they contained information on the impact of the intervention. These included those using experimental (i.e., randomized controlled trials) or observational (i.e., controlled trials without randomization, and pre-post and time series) approaches. Duplicated papers were excluded. Studies were screened for inclusion in three phases:

1. BN and FO went over all the abstracts, together, to remove the articles that certainly did not meet the inclusion criteria.
2. The full text of all the remaining abstracts were reviewed independently by BN and FO. The results were discussed. Any discrepancies were investigated and reflected upon until reaching consensus.
3. Finally, all remaining articles were thoroughly reviewed for summarizing purposes based on a preset template: research study objective, context, design, method, sample, intervention, and main conclusion.

Articles were included if:

a) Empirical/applied (i.e., theoretical studies or systematic reviews, and studies using secondary data were excluded),
b) Conducted in one or more university,
c) Aimed at evaluating, the immediate or long-term effect of an intervention on the mental health status of students,
d) Included global measures of mental health and well-being,
e) Had the university counselor involved in the intervention,
f) Involved full-time students, and
g) Was written in English.

### Quality Assessment

The quality of each of the included articles was evaluated considering the internal and external validity. For the internal validity (risk of bias), each study's methodological quality was assessed using the criteria introduced by Jadad et al. (34). As for the external/ ecological validity of the included studies, it was assessed using the criteria developed by Green and Glasgow (35). This quality assessment was not used to exclude articles. Yet, the results of the assessment were thoroughly reflected upon as an evaluative measure of the review output.

### Data Analysis

The interventions referred to in the included studies were analyzed by the researchers using the framework of Braun and Clarke (36). The intention was to inductively build a general interpretation of all included studies, in alignment with the paradigm of constructivism (37, 38). The assumption was that reality is socially-constructed. This required thoroughly reflecting upon the interventions investigated in the included studies. The process of exploratory reflection adapted was spiral, where the researchers' observations kept getting revisited which culminated into the development of an evidence-driven model. Since the constructivism paradigm gives precedence to thoroughness and insightfulness over extensiveness and generalizability (39), the decision was made upfront, as abovementioned, for this search to be limited to a single database (40). As for the purpose of the qualitative meta-synthesis, it was to create a dynamic individual-level intervention framework that is holistic and context-specific (41). All articles were categorized based on the nature of the intervention(s) under investigation. It is all narratively presented in the results section.

## Results

**Figure 1:**
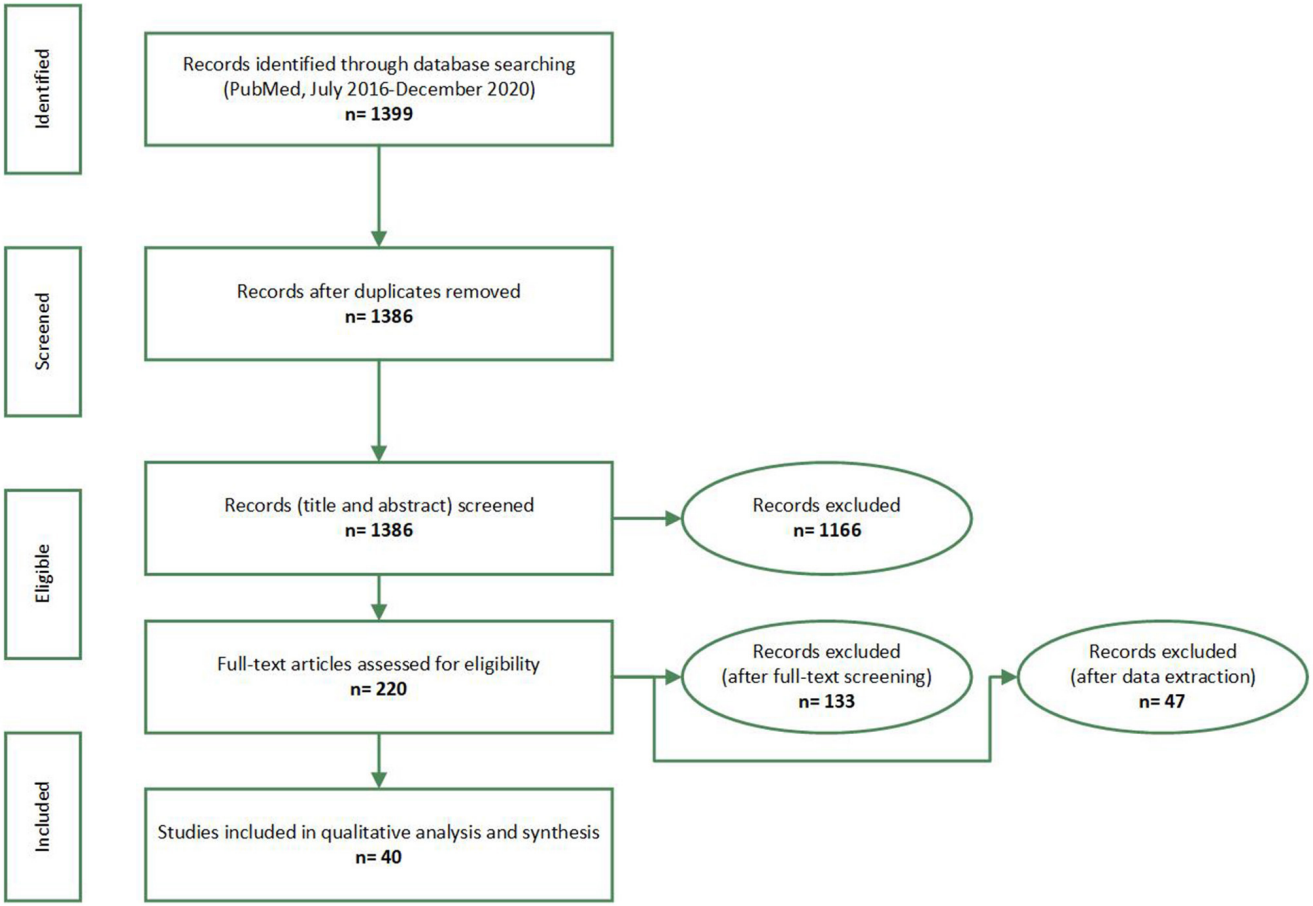
PRISMA flow-diagram. Promoting university students' mental health: a systematic literature review introducing the 4M-Model of individual-level interventions, Dubai, United Arab Emirates, 2020.

A total of 1,399 records were identified by the electronic search. Two researchers (BN and FO) reviewed all the abstracts of the resulting papers to identify ones that fitted the inclusion criteria. Based on that, a total of 1,178 articles were excluded. The full text of all remaining 220 articles were extracted and thoroughly reviewed by the two researchers (110 by each). Accordingly, 133 articles were excluded. The remaining 87 articles underwent another round of assessment by both researchers together. Out of these 87 articles, 47 papers were excluded: four studies did not meet the eligibility criteria of having an intervention in them, 31 studies did not include assessing the effectiveness of an intervention,10 studies were not exclusively on university students, and 1 was not on full-time students. Also, one study was excluded because it was not counselor-led but outsourced. Out of the initially identified 1399 articles, 40 articles were finally included in the study (Figure 1).

Of the 40 studies, nine studies were conducted in USA, eight in United Kingdom, four in Canada, three in Australia, five in Germany, four in China, and one in each of Turkey, Hungary, Israel, Ireland, Japan, South Korea and Netherlands. The quality of evidence is very high in terms of internal validity because most of the studies (25) employed RCT, five studies used a quasi-experimental method, two had a cross sectional design, and eight studies utilized a pre-post design without a control group.

The external validity of the papers could be considered low/ moderate. Since most of the studies indicated the experience of only one institution; generalization of the findings is limited. The only exceptions were one study that was conducted in Israel which included three institutions and one conducted in UK which included eight universities. After thoroughly reflecting upon the interventions under investigation across all 40 resulting studies, the authors qualitatively synthesized a holistic framework. This involved inductively identifying four overlapping categories of interventions. Each category was in turn coded with a label that appeared to be most fit to the encapsulated interventions and that is in harmony with the codes of the rest of the categories (i.e., alliteration).

Accordingly, each study was linked to at least one of four overlapping categories based on the nature of the intervention(s) under investigation (Table 1). The first category, coded as Mindfulness, included individual-level interventions that used mindfulness as a strategy to promote mental health. Mindfulness, in this context, refers to any intervention that aims to promote living in the moment or “now” and adopting acceptance and a non-judgmental attitude to guide action. The popular Mindfulness Based Stress Reduction (MBSR) curriculum was used in four studies (8, 42–45). Mindfulness Based Cognitive Therapy (MBCT) which focuses on reframing thoughts along with becoming aware of the nature and quality of them was found to also be effective in two studies (46, 47). In three studies, the intervention(s) made use of imagery and self-guidance (48–51), whereas two other studies explored the effectiveness of Acceptance and Commitment Therapy (ACT) (6) to improve the psychological flexibility, school engagement, and mental health among University students.

**Table 1:** Distribution of the output of the systematic literature review depending on the nature of the intervention(s) under investigation.

**Combinations**				**Number of occurrences/combination**
**Mindfulness**	**Movement**	**Meaning**	**Moderator**	
+	+	+	+	1
+	+	+	–	3
+	+	–	+	1
+	–	+	+	3
–	+	+	+	1
+	+	–	–	9
+	–	+	–	3
+	–	–	+	3
–	–	+	+	4
–	+	–	+	1
–	+	+	–	2
+	–	–	–	0
–	+	–	–	0
–	–	+	–	7
–	–	–	+	2
Total number of occurrences				40

*Promoting university students' mental health: a systematic literature review introducing the 4M-Model of individual-level interventions, Dubai, United Arab Emirates, 2020*.

The second category of studies was coded as Movement and included individual-level interventions which have a predominant physical element and solicit change in bodily sensations including but not limited to yoga, fitness, dance, kickboxing, and aerobics and breathing exercises. While Tong et al. (52) exclusively looked at the effect of Yoga and Fitness on mental health, five sets of researchers (8, 42, 43, 45, 46) looked at breathing and simple yoga as part of their mindfulness course. Sleep was studied in connection to mental health in two studies (53, 54) as it has been found to be a precursor to many mental health problems with insomnia and the quality of sleep put on top of the list affecting sleep hygiene. Behavioral activation, a personalized therapeutic tool mainly used in the treatment of depression targeting behaviors that feed into the condition, was found to be effective in three studies that were reviewed (55–57) involving students with mild depression. The goal of Behavioral Activation is engaging in enjoyable activities with a part of the process focusing on getting past obstacles that may impede that enjoyment. One study included peer-led support (56) and online delivery of the course (57), where both appeared to be efficacious. Only one study by Chalo et al. (58) used Biofeedback intervention, that involved measuring students' quantifiable bodily functions to convey information to them in real-time as a solution to help students manage their physiological response to anxiety and stress.

The third category was coded as Meaning and included studies that investigate individual-level interventions that focus on the counselor addressing connections and associations between variables and enabling the student to reframe cognitions. Psychoeducation was widely utilized with cognitive training as the most common (54, 59–63). Eustis et al. (49) focused their study on the student's self-awareness, while Demir and Ercan (64) explored communication techniques among students. In addition, three studies explored the feasibility of having courses embedded within the curriculum (38, 48, 50) to improve the mental health of students, while nine studies explored the effect of elective courses that aimed at stress reduction (18, 43, 50, 56, 58, 65–69).

The last category of studies was coded as Moderator which referred to any element of support that was deployed in conjunction with the counselor, in an individual-level intervention, that acts as a moderator between the student and the counselor. Pet therapy was explored in three studies (70–72) to assess well-being, and an extensive use of the computer to deliver courses such as ACT, Psychoeducation, and Cognitive Behavior Therapy (CBT) which are all traditionally effective in psychotherapy, were found to be efficacious online in 10 studies (44, 50, 57, 61, 73–78) highlighting the significance of the potential of web-based interventions to impart psychotherapy to a wider audience.

**Figure 2:**
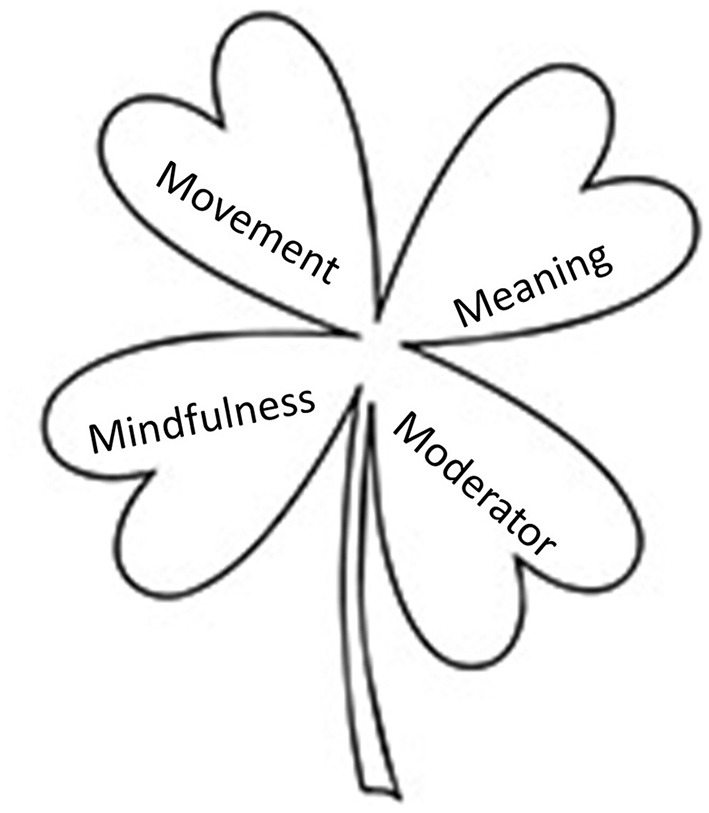
The 4M-Model generated from this study's qualitative synthesis, visually illustrated as a four-leaf clover which is a symbol of luck. Promoting university students' mental health: a systematic literature review introducing the 4M-Model of individual-level interventions, Dubai, United Arab Emirates, 2020.

This literature review showed that elements of Mindfulness were a major part of the 23 studies, Meaning was predominant in 24 studies, while Movement was an important feature in 17 studies. An element of support complementary to the therapist, either in the form of a pet (canine) or a web/phone application (i.e., Moderator), was part of 16 interventions. Commonly used approaches were Mindfulness based therapies, ACT, Cognitive Behavior Therapy, and Psychoeducation. The duration of the interventions investigated in the included studies ranged between 1 and 12 weeks, with most of the studies spanning between 6 and 8 weeks. Nine studies had just one element, and only one study (49) had all the four elements included (Figure 2), which the authors perceived as a “lucky find.”

Thirty-one studies had overlapping elements indicating that these elements are not mutually exclusive and rather interlinked and are blended with the intention of enhancing the effectiveness of a program.

## Discussion

The output of this Systematic Literature Review revealed diverse interventions. Most of these interventions were hybrid versions of existing evidence-based interventions. A few of the identified articles reflected upon contextualized home-grown interventions. There appeared to be a lack of consensus on a common model/ approach to effectively improve the mental health and wellness of university students (61) who are known to have their own set of challenges. Hence, this paper provides an outline of practices that have been deployed in this direction, illustrating them from a holistic perspective. Elements of mindfulness, meaning, movement, and use of a moderator were seen to overlap in the studies. The blending of these elements was proven to be effective in improving metacognitive awareness, emotional regulation (79), concentration, and mental clarity (80), and decreasing emotional reactivity (81) and rumination (through disengagement with persistent negative thoughts) (82) and in turn reducing depression, stress, and anxiety (83). It has also shown to foster social connectedness and the ability to express oneself in various social situations (84) thereby reducing stress and anxiety and increasing patience, gratitude, and body awareness (85). With so many elements that need to be taken into consideration, the researchers have attempted to comprehend the output of this review from the field theory point-of-view where the “organism and environment are perceived as part of an interacting field” (86).

Moreover, Counseling strategies and interventions are meant to emphasize on the growth of an individual. The human potential for self-actualization, a concept understood by Abraham Maslow as a change process that aims at making a person “aware of what is going on inside himself” [Maslow, as cited in Seaman (87), p. 3] is core to Counseling interventions, which is where the four elements blend to become crucial to the process of self-awareness and eventually self-growth.

The results of the study indicate that self-awareness through mindfulness is an important foundation upon which all other elements build up to improve mental health of students. This was not a surprising find because this is in alignment with the results of many previously conducted studies (88, 89). Mindfulness seems to be the new mantra and has been intensively researched (90). However, despite a substantial amount of theoretical work conducted to merge Buddhist and Western conceptual viewpoints to psychotherapy (91), there is minimal literature on how it can translate to practice making this review an important addition to the limited knowledge around the topic of psychological interventions that have been found to be effective among university students. MBSR has proven to reduce stress and anxiety among university students by fostering insight and concentration along with physiologic relaxation (92). Teaching students to live in the present moment by reframing thoughts (i.e., MBCT) has been found to be effective in reducing depression (93). It also lessens the risk of relapse with comparable efficacy to antidepressant medication (94) which, in itself, is a breakthrough for psychotherapy. ACT which focuses on acceptance has been found to improve coping, self-regulation, psychological flexibility, and school engagement (6). Counseling young adults, in particular students at the university level, would benefit by basing it on Engel's biopsychosocial viewpoint which includes taking into consideration the hormonal changes (biological), identity crisis, and the challenges arising from intimacy and isolation (psychological) which have been hypothesized in Eric Erickson's psychosocial stages of development for this age group. The new age technological challenges of peer-pressure over social media sites and the demands of fitting in and changing family dynamics (sociological) also need to be taken into consideration when conceptualizing a Counseling program for this target group.

Moreover, this transition stage between adolescence and adulthood, also referred to as “emerging adulthood” (95), is considered to be a period of accepting responsibility for one's actions and livelihood, developing belief systems and values independent of parental and external influences, and establishing relationships with parents on equal grounds. Young university students who are still financially dependent and living with parents during this period are arbitrarily considered to be adolescents if adult responsibilities are not yet accessed. These intangible markers gradually develop. The entailed process could last many years until the corresponding responsibilities are effectively adopted. As such, the range between adolescence and adulthood becomes wider than typically defined, stretching from the beginning of puberty to the early twenties (96).

Counseling has been traditionally associated as a profession that requires the physical presence of a minimum of two people in a professional relationship to talk through and process experiences to gain insight and understanding. However, in this review, it is evident that web-based interventions seem to produce an equally effective result (97) as observed in 16 studies of the literature review which could be utilized as a complementary medium widening the scope of practice of counselors and psychotherapists. This could also help in minimizing the stigma associated with getting undesirably labeled and help in reducing psychological self-restraint which has been termed as ‘online disinhibition effect' (98). Web-based mental health interventions also are becoming a preferred medium for students to gain services and information (99) as they accommodate their busy schedules (100).

Another observation was that even though most of the interventions were conducted only for a short period of time, the effectiveness of the interventions was established. Embedding interventions within the curriculum has been suggested (101) which makes this review even more pertinent for innovations in curriculum planning. This may also help in alleviating the stigma that is attached to Counseling services which is often a barrier that prevents students from reaching out for help (102). This aligns with Vygotsky's notion of Zone of Proximal Development (103) which refers to pedagogical support being beneficial for activities, in this context, psychoeducation of positive behaviors that facilitate help seeking behaviors before they can start using them independently.

The above observations prompted the researchers to recognize that the four identified elements when combined would result in a holistic approach of addressing the individual from a biopsychosocial point-of-view. This was depicted in the form of the 4M-Model to guide counselors to develop and implement university-level interventions that could help to reduce stress, anxiety, and depression as well as improve emotion regulation and self-awareness to address the mental health needs of young adults. It would be worthwhile for future research studies to validate the suggested 4M-Model through a similar systematic review of the literature relying on a combination of databases (104). The analysis in this case would be deductive where the model conceived from this study can be used as a preset template. Also, for validation purposes, it is recommended to conduct follow-up studies aimed at evaluating the efficaciousness of a tailor-made assortment of interventions that can be linked to all elements of the 4M-Model. For that purpose, it would be useful to adapt a mixed methods approach to research, where quantitative and qualitative findings will be integrated to obtain a holistic perspective of the output, outcome, and impact of such university-based, individual-student level mental health initiatives.

## Conclusion

Findings of this review reveal the 4M-Model that happen to address all aspects of holistic well-being: physical, psychological, emotional, and social. Effectiveness of the varied interventions that have been reviewed in this study indicate that if a comprehensive approach toward intervention including mindfulness, movement, moderator, and meaning is adapted, then it would not only help students to be supported in a holistic manner but would help counselors plan and execute their programs in a focused approach to address the needs of any university student population who are increasingly overwhelmed and burned out with the stressors from their outside worlds as well as from within. The findings from the review add to the growing evidence for the urgent need of an intervention model that can serve as a directive for counselors and students.

## Data Availability Statement

The original contributions presented in the study are included in the article/supplementary material, further inquiries can be directed to the corresponding author/s.

## Author Contributions

BN and FO conceptualized the study, conducted the review, performed the qualitative meta-synthesis, and prepared and approved the manuscript. Both authors contributed to the article and approved the submitted version.

## Conflict of Interest

The authors declare that the research was conducted in the absence of any commercial or financial relationships that could be construed as a potential conflict of interest.
